# Willingness to pay for flexibility at the workplace for people with diabetes and chronic disease: a discrete choice experiment in a population of workers in Denmark

**DOI:** 10.1186/s12889-019-6919-6

**Published:** 2019-05-16

**Authors:** Kristoffer Panduro Madsen, Bryan Cleal, Kasper Olesen, Lise Hagelund, Ingrid Willaing

**Affiliations:** 1 Diabetes Management Research, Steno Diabetes Center Copenhagen, Niels Steensens Vej 6, DK-2820 Gentofte, Denmark; 2 Incentive, Holte, Denmark

**Keywords:** Diabetes mellitus, Workplace, Flexibility, Willingness to pay, Discrete choice experiment

## Abstract

**Background:**

The number of people of working age suffering from chronic disease is increasing. Chronic diseases such as diabetes can cause negative work-related consequences in the form of early retirement or absenteeism. Providing flexible workplace accommodations may enable the person with diabetes to retain their position in the labor market. However, the successfulness of such accommodations depends largely on the perceptions of those not suffering from diabetes. The purpose of this study was to examine preferences of a population of workers in Denmark for flexibility at the workplace, for people with diabetes and for people with chronic disease in general, measured as their willingness to pay (WTP).

**Methods:**

Respondents were drawn from online panels and randomized to answer an online survey regarding flexibility at the workplace for people with diabetes or chronic disease in general. One thousand one hundred and three respondents were included in the analysis. Based on discrete choice experiments included in the survey, we analyzed WTP for five flexibility attributes: part-time, customizing job description, additional break with pay and time off for medical visits with and without pay. We further examined perceptions of the employer’s responsibility to ensure workplace flexibility for five different specific chronic diseases including diabetes. Finally, we analyzed differences in WTP for flexibility across subgroups.

**Results:**

Respondents’ WTP was significantly higher for chronic disease in general compared to diabetes for the possibility of part-time (81€/month vs. 47€/month, *p* < 0.001) and customizing job description (58€/month vs. 41€/month, *p* = 0.018) attributes, as well as for the overall average (49€/month vs. 36€/month, *p* = 0.008). Ensuring workplace flexibility for patients with a specific chronic disease other than diabetes (cancer, heart disease, arthritis and COPD) was to a higher degree considered a responsibility of the employer. Average WTP for flexibility varied across subgroups, consistently yielding a larger amount for chronic disease in general.

**Conclusions:**

The population examined in this study are willing to pay less for flexibility at the workplace for people with diabetes compared to people with chronic disease in general. This finding was evident in terms of specific flexibility attributes and on average across subgroups.

**Electronic supplementary material:**

The online version of this article (10.1186/s12889-019-6919-6) contains supplementary material, which is available to authorized users.

## Background

The global increase of people with diabetes and other chronic diseases is generating new challenges for health care systems across the globe [1]. With increased longevity, governments around the world have sought to increase the statutory age of retirement and thereby the average age of the workforce. One inference to be drawn from this is that the proportion of individuals in the workforce diagnosed with one or more chronic health conditions will also increase [2]. Given this, it is important to direct increased attention to what is required to enable people living with chronic conditions to retain their position in the labor market. Numerous studies have shown that having a chronic disease such as diabetes, cancer, heart disease, arthritis, and chronic obstructive pulmonary disease (COPD) increases the risk of work disability [3–7]. Indirect consequences of working while managing a chronic disease may also take their toll. People with a chronic disease are, for example, more prone to experience elevated levels of work-related fatigue [8], and this is a strong predictor of work disability [9, 10].

Flexibility at the workplace has been suggested as a means to avoid productivity loss due to early retirement and absenteeism [11, 12]. Studies have shown that providing flexible working conditions to employees can lead to increased engagement, motivation, job satisfaction and a better work-family balance [13, 14]. Other research has shown that flexible working conditions lead to reduced stress and burnout and to better physical health [15, 16]. Flexibility in the context of work can take many guises, but when considering the work-specific challenges confronted by people living with chronic disease, tailored accommodations which make the workplace a more amenable place can, potentially, play an important role in helping people remain productive at work [12, 17]. The degree to which such accommodations are likely to be implemented and successful will, however, be partly determined by the extent to which they are recognized as effective and appropriate by those who do not suffer from chronic disease. At present, knowledge about preferences and attitudes regarding the perceived efficacy and suitability of workplace accommodations is sparse.

In recent years there has been an increased use of stated preference methods in which willingness to pay (WTP) is used to elicit health related preferences [18, 19]. WTP is defined as the maximum amount of money a person is willing to pay for a certain good or service. In this study we investigated the WTP of a population of workers in Denmark without diabetes for flexibility at the workplace for people with diabetes, brought into relief by comparison with WTP for flexibility at work for people with chronic disease in general. Furthermore, we analyzed the opinion of the employer’s responsibility to ensure flexible working conditions for people with cancer, heart disease, arthritis, COPD and diabetes. Finally, we analyzed WTP for flexibility at the workplace for diabetes compared to chronic disease in general across subgroups.

## Methods

### Data collection

This study is based on data (*n* = 1200) acquired in larger a survey conducted in Denmark in the spring of 2015 from March 24 to June 10. The survey was performed as an online survey and respondents were recruited by e-mail from existing online panels with more than 150,000 Danish panelists (http://www.userneeds.dk). Weights based on age, gender and geography were applied to reflect the demographic pattern of the working-age population. Inclusion criteria were age between 25 and 67 years, employed in a place of work with at least one colleague, and residence in Denmark. The overall focus of the larger survey was diabetes in work life and for this reason people with diabetes were oversampled in the recruitment process. Results from the larger study, including people with diabetes, have previously been published elsewhere [20]. For the purposes of this study, however, in which the primary focus are the preferences of a working population without diabetes in Denmark, people with diabetes were excluded from the analysis.

### Survey instrument

The survey included items regarding participant demographics such as age, gender, education and employment status. The survey also included items regarding individuals’ health status and a set of discrete choice experiments (DCEs) designed to measure participants’ WTP. In a DCE, individuals are asked to choose between a set of hypothetical scenarios which vary across different attributes and levels, incorporating a hypothetical monetary attribute, making an analysis of WTP for each attribute possible [21]. This approach quantifies the respondents’ preferences through their WTP, under the assumption that the chosen scenario represents their preferred option. We assessed WTP for flexibility at the workplace for five attributes; possibility of part-time, possibility of customizing job description, additional break with pay, and time off for medical visits and education with and without pay (Table 1). The underlying premise was that respondents were to imagine that the amount they were willing to pay would be subtracted from their monthly pay check after tax (see Additional file 1 for the exact wording used to introduce the DCEs). Upon initiation of the survey, we allocated 50% of the respondents to answer the DCEs with regards to people with diabetes and 50% with regards to people with chronic disease in general. In addition to the DCEs, the survey also contained questions regarding perceived degree of the employer’s responsibility in ensuring flexibility at the workplace for people with cancer, heart disease, arthritis, COPD and diabetes.

**Table 1:** Work-related flexibility attributes at the workplace

Attribute	Level
Possibility of part-time	Yes
	No
Possibility of customizing job description	Yes
	No
Additional break with pay	Yes
	No
Time off for medical visits and education	Yes – with pay
	Yes – without pay
	No
Reduction in monthly pay check after taxes	DKK 50 / 6.6 €^a^
	DKK 100 / 13.3 €^a^
	DKK 200 / 26.6 €^a^
	DKK 500 / 66.6 €^a^

^a^Conversion from DKK (Danish Krone) to € is based on the average exchange rate in 2015 of 7.45

### Design of discrete choice experiments

The face validity of the attributes and levels used to design the DCEs was based on expert opinions and literature review. Initially these were tested at a diabetes clinic in Denmark with six people with diabetes. These individuals were subsequently interviewed about their opinions regarding the relevance of the attributes and levels and the extent to which they found the questionnaire to be usable and comprehensible. Finally, a pilot test among 92 individuals from online panels led to an increase in the levels of monthly pay reductions as they were initially set too low.

The attributes and levels in the DCEs could be combined in many ways. To reduce the number of questions to a manageable size, we used a standardized process in Ngene®. The design practices used here are in line with the practices described in Johnson et al. [21]. Twenty-four DCE scenarios focused on work-related flexibility were generated (see Table 2 for an example of a scenario). The scenarios were divided into 4 groups and randomized into 4 different versions of the questionnaire. Thus, the individual respondents were presented with 6 scenarios regarding flexibility at the workplace for which their WTP for each preferred option could be obtained. Table 2 presents an example (see Additional file 2 for a complete 6-scenario combination) of one of the scenarios in which the respondents were asked if they preferred the combination of attributes in option A or option B while simultaneously considering the given WTP amount. Answering multiple scenarios permits estimation of a population-average WTP for each attribute. If respondents reported that they did not understand the scenarios or gave conflicting responses, they were excluded from the analysis.

**Table 2:** Example of a discrete choice experiment for work-related flexibility

Attribute	Option A	Option B
Possibility of part-time	No	Yes
Customizing job description	No	Yes
Additional break with pay	Yes	No
Time off for medical visits and education	Yes – the person can attend with pay	No
Reduction in pay check after taxes per month	DKK 200 / 26.6 €^a^	DKK 100 / 13.3 €^a^
Which option do you prefer?	□	□

^a^Conversion from DKK (Danish Krone) to € is based on the average exchange rate in 2015 of 7.45

### Statistical analysis

The discrete choice responses were analyzed using the conditional logit model using a procedure previously described [18, 20]. We undertook a bootstrapping exercise using 10,000 iterations as recommended in Barker [22] to compute confidence intervals for the WTP estimates. To analyze differences in respondent characteristics, we used Pearson’s Chi-squared test and Fisher’s Exact test for categorical variables and Student’s t-test for continuous variables. All *p*-values were two-sided and values < 0.05 were considered statistically significant. All statistical analyses were performed using SAS version 9.4.

## Results

### Respondents

In total, 1139 respondents met the inclusion criteria and completed the questionnaire relevant for this study. Thirty-six respondents were excluded because they reported not understanding the DCE scenarios rendering a study population of 1103 participants consisting of 534 (48%) women and 569 (52%) men with an average age of 45. There were no statistically significant differences in respondent characteristics between the group asked about diabetes and those asked about chronic disease in general (Table 3).

**Table 3:** Respondent characteristics

N	Respondents asked about diabetes (%)	Respondents asked about chronic diseases (%)	Difference *P*-value
	540	563	
Men	272 (50)	297 (53)	
Women	268 (50)	266 (47)	0.429
Age (average)	46 (SD = 10.38)	45 (SD = 10.43)	0.450
Full-time employment	515 (95)	541 (96)	
Part-time employment	25 (5)	22 (4)	0.553
Public sector	214 (40)	226 (40)	
Private sector	326 (60)	337 (60)	0.862
More than 3 years of further education	303 (56)	315 (56)	
Less than 3 years of further education	237 (44)	248 (44)	0.957
Relatives or friends with diabetes	205 (38)	184 (33)	
No relatives or friends with diabetes	335 (62)	379 (67)	0.067
Arthritis	30 (6)	23 (4)	0.254
Asthma	18 (3)	23 (4)	0.509
Back pain	19 (4)	27 (5)	0.289
Depression	22 (4)	24 (4)	0.875
Decreased hearing	12 (2)	11 (2)	0.755
Migraine	29 (5)	23 (4)	0.314
Other long-term disease	52 (10)	54 (10)	0.983

Reported chronic conditions had been treated within the past year upon completion of the survey. Differences tested with Chi-squared tests and Student’s t-test for categorical and continuous outcomes, respectively

*SD* Standard deviation

### Preferences for flexibility at the workplace

**Fig. 1:**
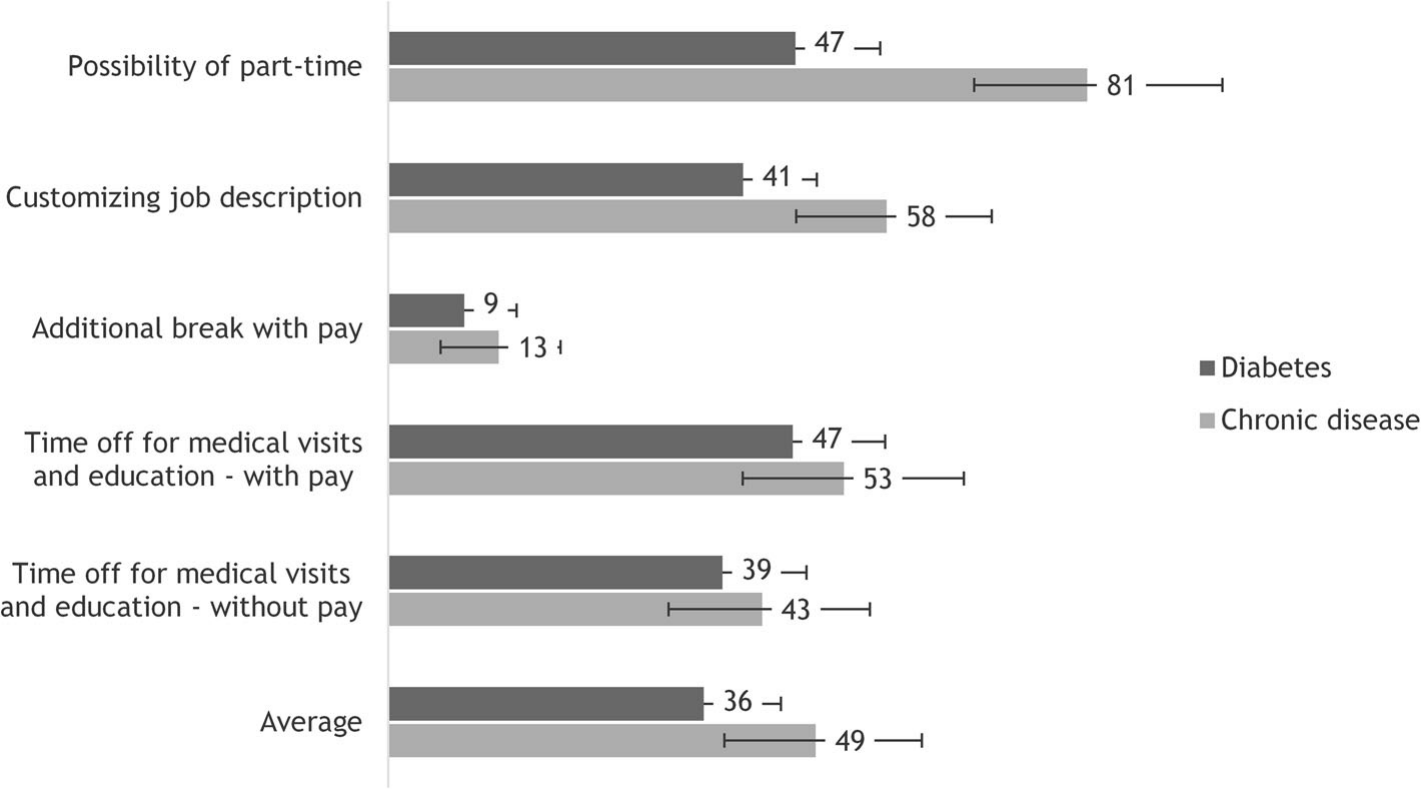
Willingness to pay for flexibility attributes at the workplace, €/month. The error bars represent 95% confidence intervals

There was a WTP for flexibility at the workplace both for diabetes and chronic disease in general, with the latter having a higher WTP across all attributes. WTP was significantly higher in relation to chronic disease in general compared to diabetes for the *Possibility of part-time* (*p* < 0.001) and the *Customizing job description* (*p* = 0.018) attributes (Fig. 1). Respondents asked about flexible working conditions for diabetes were willing to pay 47 € per month [CI (95% confidence interval): 39–57 €] for the possibility of part-time, while respondents asked about flexible working conditions for chronic disease in general were willing to pay 81 € per month [CI: 68–96 €]. With regards to the possibility of customizing job description, respondents asked about diabetes were willing to pay 41 € per month [CI: 33–49 €] and respondents asked about chronic disease in general were willing to pay 58 € per month [CI: 47–70 €]. The difference in average WTP across all attributes was also statistically significant between the two groups (*p* = 0.008) with 36 € per month [CI: 28–45 €] for people asked about diabetes compared to 49 € per month [CI: 28–60 €] for people asked about chronic disease in general.

### Employer responsibility for flexible working conditions

**Fig. 2:**
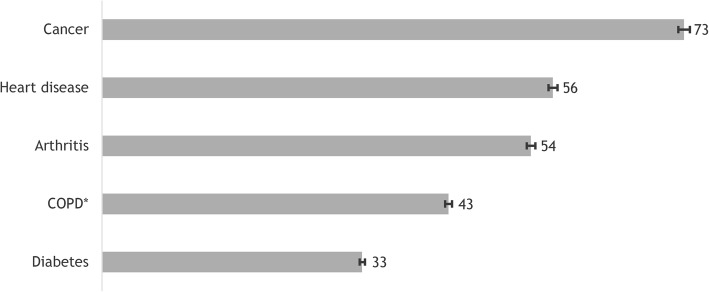
Share (%) that think it is to *a large or very large degree* the employer’s responsibility to ensure flexible working conditions for people with different chronic diseases. The error bars represent 95% confidence intervals. *Chronic obstructive pulmonary disease

When asked about employer responsibility for flexible working conditions for specific chronic diseases, there were clear differences in opinions regarding cancer, heart disease, arthritis COPD and diabetes, respectively (Fig. 2). When asked about cancer, 73% [CI: 70–75%] thought it was to a large or very large degree the employer’s responsibility to ensure flexible working conditions. In the case of heart disease and arthritis, 56% [CI: 53–59%] and 54% [CI: 51–57%], respectively, found the employer to be responsible for ensuring flexible working conditions. This was the case for 43% of respondents [CI: 40–46%] regarding COPD and for 33% [CI: 30–35%] of respondents regarding diabetes.

### Differences in flexibility preferences across subgroups

Table 4 shows the average WTP for flexibility across different subgroups. There were statistically significant differences in WTP among women (*p* = 0.012), people aged 25–49 years (*p* = 0.042), people with less than 3 years of further education (*p* = 0.012), people who do not have a relative with diabetes (*p* = 0.028) and people who themselves had been treated for a chronic disease other than diabetes within the last year (*p* = 0.012).

**Table 4:** Willingness to pay for flexibility at the workplace for people with diabetes and chronic disease in general among subgroups, €/month deducted from pay-check after taxes

	N (%) Diabetes	N (%) Chronic disease	WTP Diabetes (€)	WTP Chronic disease (€)	Difference *P*-value
Average	540	563	36	49	0.008^*^
Gender					
Male	272 (50)	297 (53)	44	55	0.248
Female	268 (50)	266 (47)	32	46	0.012^*^
Age					
25–49 years	316 (59)	338 (60)	32	43	0.042^*^
50–67 years	224 (42)	225 (40)	45	69	0.080
Education					
Less than 3 years of further education	237 (44)	248 (44)	34	54	0.012^*^
More than 3 years of further education	303 (56)	315 (56)	39	47	0.224
Relative with diabetes					
Do not have relatives or friends with diabetes	335 (62)	379 (67)	39	55	0.028^*^
Have relatives or friends with diabetes	205 (38)	184 (33)	34	40	0.352
Morbidity^a^					
Not treated for chronic disease within the last year	381 (71)	412 (73)	39	48	0.140
Treated for chronic disease within the last year	159 (29)	151 (27)	33	57	0.012^*^
Employer responsibility^b^					
Employer responsible for flexible work conditions	124 (23)	112 (20)	59	76	0.436
Employer not responsible for flexible work conditions	416 (77)	451 (80)	29	46	< 0.001^*^

^a^Diseases – arthritis, asthma, atherosclerosis, back disease, cancer, COPD, depression, decreased hearing, migraine, stroke and other long-term disease

^b^Employer is to a large or very large degree responsible for ensuring flexible working conditions for diabetes, COPD, cancer, heart disease and arthritis. Differences tested with Chi-squared tests

^*^Statistically significant at an alpha of 0.05

People who answered that it to a large or very large degree was the employer’s responsibility to ensure flexible working conditions for all the specified chronic diseases had no difference in WTP values for diabetes and chronic disease in general. People who answered that it was not the employer’s responsibility to ensure flexible working conditions for at least one of the chronic diseases were willing to pay more for flexible working conditions for people with chronic disease in general than for people with diabetes (*p* < 0.001).

## Discussion

The population examined in this study is willing to pay more for flexibility at the workplace for people with a chronic disease in general than they are prepared to pay for people with diabetes. The difference in WTP was statistically significant for the “*Possibility of part-time”* and “*Customizing job description”* attributes, as well as for the overall average. There were also statistically significant differences in WTP between diabetes and chronic disease in general across subgroups, all of which were found to be higher for chronic disease. This was evident for the subgroups: female, 25–49 years, less than 3 years of further education, relatives or friends with diabetes, treated for a chronic disease within the last year, and the employer not being responsible for flexible working conditions. Our results further indicate that people perceive diabetes, in relative terms, to be of least importance when considering the extent to which employers should ensure flexible work conditions for people with chronic diseases. Although certain flexibility attributes and subgroup analyses did not reach statistical significance, possibly due to sample size, the estimated WTP amounts still indicate an empirically relevant WTP in both groups.

The differences in WTP and opinions regarding employer responsibility may be explained by the perceived severity of the diseases in question. Previous studies have shown that diabetes is perceived as the least severe disease in comparison with heart disease, cancer and stroke [23–25]. Furthermore, it has been shown that people view diabetes as a relatively controllable disease as compared to cancer for example [23, 24]. Other research reveals that concern about developing diabetes is relatively low for both women, for whom concerns about breast cancer and heart disease figure more prominently, and men, among whom heart disease and prostate cancer give cause for greatest concern [25]. Likewise, it has also been demonstrated that people have a tendency to underestimate their risk of developing type 2 diabetes [26]. Viewed in light of the existing literature, our results suggest that people of working age do not regard diabetes as a disease that requires as much flexibility or accommodation in the workplace as other chronic diseases, at least in terms of their WTP. This is indicative of a prevailing perception about diabetes as a condition which is essentially manageable and thus not requiring the same level of flexibility or accommodation in the context of work as, for instance, cancer and heart disease. This suggests a potential need for dissemination of knowledge on how to support people with diabetes to be able to reconcile diabetes and work life and to enable people with diabetes to stay in the labor market without limitations brought on by their condition.

Our analysis of WTP for diabetes and chronic disease in general across subgroups revealed that female participants were willing to pay significantly more for chronic disease in general compared to diabetes, while no differences were found in the male subgroup. Being in the younger subgroup and being in the subgroup with a lower level of education also yielded significantly higher WTP values for chronic disease in general compared to diabetes. One potential reason for these observations is the relationship between the respective subgroups and their mean income. All three subgroups, at least in Denmark, are known to have a lower income compared to their counterparts (males, older age group, more than 3 years of further education) [27, 28]. Previous research has shown that income is positively correlated with WTP [29–31]. As such, the lower WTP for diabetes compared to chronic disease in these subgroups may reflect a certain difference in ability to pay or at least a different prioritization of disposable income. This inference is also supported by the fact that all three subgroups had a lower WTP value for both diabetes and chronic disease compared to their counterparts, except in the case of chronic disease in the less than 3 years of further education subgroup. WTP in different subgroups of the population may also be partly determined by risk of developing a chronic disease, e.g. indicated by the markedly higher WTP for both diabetes and chronic disease in general of the older age group compared to the younger.

The subgroup analysis further showed that people who do not have relatives or friends with diabetes are willing to pay significantly more for chronic disease in general compared to diabetes, whereas for those with a relative or friend with diabetes there was no statistically significant difference. This finding indicates that respondents with first-hand knowledge of diabetes are more inclined to recognize the need for workplace accommodations and less inclined to view diabetes as of less importance than chronic disease in general. However, the WTP value for diabetes in this subgroup was still lower than for respondents without a friend or relative with the condition. The relatively low WTP among this group may initially seem counter-intuitive, suggesting a perception of diabetes as a relatively manageable disease among people who know someone with the condition. At the same time, in the context of the Danish welfare state, it may also reflect the fact that people who are familiar with diabetes feel that workplace accommodations should be covered by existing legislation regarding flexibility at the workplace.

The respondents who had themselves been treated for a chronic disease within the past year reported a significantly higher WTP for chronic disease in general compared to diabetes, while not having been treated resulted in no statistically significant difference. While not a surprising finding, this result indicates that people who have first-hand experience with chronic disease value flexibility at the workplace higher for chronic disease in general than for diabetes and, since this outcome is to be expected, supports the validity of the discrete choice methodology as capable and sensitive enough to ascertain actual, true preferences.

The results we present indicate that diabetes has a relatively modest ranking as a condition for which flexibility and accommodation at work are perceived to be justified. Public perceptions about diabetes in this context do not, therefore, tally well with what is known about the demands and consequences of the condition in the context of work life [32]. Numerous studies have demonstrated that a diagnosis with diabetes impacts negatively on a range of labor market outcomes e.g. early retirement [33], productivity [34], absenteeism [35] and income levels [36]. The findings we present here indicate that the working population is willing to pay for flexible working conditions for people with chronic disease in general as well as for people with diabetes. However, diabetes was across all our results consistently rated as a disease requiring less flexibility at the workplace compared to chronic disease in general, indicating a lack of knowledge and understanding about the actual scale of the problem of having diabetes in the context of work life.

### Strengths and limitations

To our knowledge, this is the first study comparing WTP for flexibility at the workplace for people with diabetes and for people with chronic disease in general. Strengths of this study include the large study population and high response efficiency. Furthermore, there were no statistically significant differences in demographic or chronic disease variables between the group asked about diabetes and the group asked about chronic disease in general, limiting bias arising from heterogeneity between groups. A noteworthy strength of the DCEs used in this study is the ability to concretize the rather abstract question of how much one is willing to pay for a hypothetical attribute by obliging the respondent to choose between predefined options. Another strength of the DCE is the balanced and orthogonal design resulting in a perfectly efficient design [21].

Although the DCE has many advantages, there may be some methodological limitations. DCEs may be cognitively challenging for some people, in part due to possible fatigue from the large number of questions and also due to respondents’ evaluations regarding the hypothetical context of the experiment [21]. Our response efficiency was, however, high with only 36 respondents excluded due to not understanding the DCE scenarios, indicating that the experiments were meaningful to the participants. Respondents with diabetes were excluded in this study. This may have resulted in slightly overestimated WTP values for flexible accommodations for people with diabetes at work as a previous study, surprisingly, showed relatively lower WTP for flexibility accommodations at work for people with diabetes among people with diabetes themselves [20]. Thus, the difference in WTP for people with diabetes and other chronic diseases may be even bigger than this study suggests. Furthermore, there may be issues of generalizability as it may only be a certain selected group of the general population (e.g., blue-collar workers) who participate in online surveys.

We recognize that, in seeking to set perceptions about diabetes into relief, we have compared diabetes to a number of chronic conditions with which it is often comorbid. The relative influence of diabetes specific morbidity and comorbidity in relation to the labor market outcomes of people with diabetes is, however, an important point of focus and one which research has only recently begun to address [37]. In contrast to public perceptions about the relative severity of diabetes in the context of work life, epidemiological evidence indicates a profound problem impacting both individuals and society at large. There is, moreover, the threat that diabetes will become more prevalent in the working population in the future if population ageing and lifestyle trends continue their current course. Now may be the time to take seriously the challenges that people with diabetes face in their work-life context.

## Conclusions

WTP for flexibility at the workplace was significantly higher for people with chronic disease in general compared to people with diabetes. Furthermore, employers’ responsibility for workplace flexibility in relation to diabetes was ranked lower than it was for cancer, heart disease, arthritis and COPD. WTP differed considerably across subgroups, indicating a higher WTP for chronic disease in general across all groups. These findings suggest a perceptual lacuna regarding the actual challenges faced by the individuals with diabetes in the context of work life and, moreover, to the challenges faced by society at large in terms of increasing indirect costs associated with diabetes. This suggests a potential need for dissemination of knowledge on how to support people with diabetes to be able to reconcile diabetes and work life and to enable people with diabetes to stay within the labor market without limitations brought on by their condition.

## Additional files

Additional file 1: Introduction to discrete choice experiments. The exact wording shown to participants prior to the discrete choice experiments. (PDF 205 kb)

Additional file 2: Discrete choice experiments. Example of a 6-scenario DCE combination shown to participants. (PDF 104 kb)

## Abbreviations

COPD: Chronic obstructive pulmonary disease
DCE: Discrete choice experiment
WTP: Willingness to pay
