# Supplementary material for: Willingness to pay for flexibility at the workplace for people with diabetes and chronic disease: a discrete choice experiment in a population of workers in Denmark
Source: BMC Public Health. 2019 May 16;19:584. doi: 10.1186/s12889-019-6919-6 (PMC6521535; doi:10.1186/s12889-019-6919-6)
Supplement: Supplementary file 1 — Introduction to discrete choice experiments. The exact wording shown to participants prior to the discrete choice experiments. (PDF 205 kb) [file 12889_2019_6919_MOESM1_ESM.pdf]

# Introduction to the discrete choice experiment (DCE) survey instrument:

**Overview of attributes shown to the participants prior to the DCE:**

| **Attributes** | **Wordings presented to participants** | **Attribute levels** |
|---|---|---|
| | General introduction: *'If an individual develops diabetes [chronic disease] it can be challenging if there is a lack of flexibility at the workplace. The individual may, for example, have an increased need to attend medical appointments or take unscheduled breaks and certain work-tasks can be difficult to manage.*<br><br>*In the following pages we describe different initiatives through which the workplace can provide flexibility if an employee develops diabetes [chronic disease]. The initiatives are as follows:'* | |
| Part-time option | *If an employee develops diabetes [chronic disease] should s/he be allowed to retain their job, but work on a part-time basis? Wages would be reduced accordingly.* | Yes/<br>No |
| Customizing job | *If an employee develops diabetes [chronic disease] and duly encounters difficulties in carrying out their work should s/he be allowed to change their job-description or be retrained for a new job?* | Yes/<br>No |
| Extra breaks with pay | *Employees with diabetes [chronic disease] may need to hold intermittent breaks. They should be allowed to do this without having their wages docked.* | Yes/<br>No |
| Time off for medical visits and education | *People with diabetes [chronic disease] should be allowed the opportunity to attend medical appointments or patient education courses during normal working-hours; Either by taking time off or as a part of their working day.* | Yes-with pay/<br>Yes-without pay/<br>No |
| Reduction in pay | *Monthly reduction in pay after taxes.* | 7 €/<br>13 €/<br>27 €/<br>66 € |

**Description of DCEs shown to participants asked about diabetes [chronic disease]:**

"*In the following you are to imagine that you are employed at in a large company where some of the employees are diagnosed with diabetes [chronic disease]. This could be your current or future place of work.*

*The company has different options of putting together initiatives that are directed at people who are diagnosed with diabetes [chronic disease]. Some initiatives cost money to implement, and you have to imagine that the presented amount would be subtracted from your monthly pay-check after taxes.*

*On each page there are two options. Choose the option that you prefer.*

*We emphasize that this is a thought experiment.*"
