# Supplementary material for: Willingness to pay for flexibility at the workplace for people with diabetes and chronic disease: a discrete choice experiment in a population of workers in Denmark
Source: BMC Public Health. 2019 May 16;19:584. doi: 10.1186/s12889-019-6919-6 (PMC6521535; doi:10.1186/s12889-019-6919-6)
Supplement: Supplementary file 2 — Discrete choice experiments. Example of a 6-scenario DCE combination shown to participants. (PDF 104 kb) [file 12889_2019_6919_MOESM2_ESM.pdf]

Example of a 6-scenario combination presented to the participants:

	Discrete Choice Experiment 1.		Discrete Choice Experiment 2.	
Attributes *(keywords)*	Option A	Option B	Option A	Option B
Part-time work	No	Yes	Yes	No
Customized work	No	Yes	Yes	No
Extra breaks with pay	Yes	No	Yes	No
Medical visits work hours	Yes, without pay	No	No	Yes, with pay
Monthly pay reduction	27 €	13 €	7 €	66 €
Preferred option (A or B)?	☐	☐	☐	☐
	**Discrete Choice Experiment 3.**		**Discrete Choice Experiment 4.**	
Attributes *(keywords)*	Option A	Option B	Option A	Option B
Part-time work	Yes	No	No	Yes
Customized work	No	Yes	Yes	No
Extra breaks with pay	No	Yes	Yes	No
Medical visit work hours	No	Yes, without pay	Yes, without pay	Yes, with pay
Monthly pay reduction	7 €	66 €	27 €	13 €
Preferred option (A or B)?	☐	☐	☐	☐
	**Discrete Choice Experiment 5.**		**Discrete Choice Experiment 6.**	
Attributes *(keywords)*	Option A	Option B	Option A	Option B
Part-time work	Yes	No	No	Yes
Customized work	Yes	No	No	Yes
Extra breaks with pay	Yes	No	Yes	No
Medical visits work hours	Yes, without pay	Yes, with pay	No	Yes, with pay
Monthly pay reduction	13 €	27 €	27 €	13 €
Preferred option (A or B)?	☐	☐	☐	☐

Note: This table solely serves the purpose of illustrating the different possible combinations of attributes and levels shown to the participants. The actual scenarios were presented each on a separate page with a layout similar to that of table 2 in the article manuscript.
